# Public–Private Partnerships for Universal Health Coverage in Sub‐Saharan Africa: A Scoping Review Protocol

**DOI:** 10.1002/hsr2.70746

**Published:** 2025-04-21

**Authors:** Godfred Otchere, Adam Fusheini, Trudy Sullivan, Robin Gauld, Erin Penno

**Affiliations:** ^1^ Department of Preventive and Social Medicine University of Otago Dunedin New Zealand; ^2^ Ministry for Social Development Wellington New Zealand; ^3^ Center for Health Literacy and Rural Health Promotion Accra Ghana; ^4^ Department of Management University of Otago Dunedin New Zealand; ^5^ Bond Business School Bond University Gold Coast Queensland Australia

**Keywords:** public–private partnerships, sub‐Saharan Africa, universal health coverage

## Abstract

**Background and Aims:**

Globally in the health sector, governments use public–private partnerships (PPPs) to make significant contributions to the development and implementation of national health policies and strategies to improve health systems and health outcomes. Following the World Health Organization's resolution on universal health coverage (UHC), healthcare PPPs have emerged as an innovative policy option, especially, for countries in sub‐Saharan Africa (SSA) to progress towards achieving UHC. Although many studies have reported the use of healthcare PPPs, it appears no review has been conducted to synthesize evidence on how it facilitates or hinders progress toward attaining UHC in the SSA context. This review seeks to fill this research gap by systematically synthesizing the literature on healthcare PPPs used in SSA and examining how various forms of healthcare PPPs affect progress towards attaining UHC in SSA.

**Methods:**

Arksey and O'Malley's guidelines for conducting scoping reviews will guide the review process. PubMed, Medline (Ovid), Global Health (Ovid), Web of Science, Scopus, and EconLit will be searched for studies published from January 2013 to December 2023. Thematic analysis of data will be employed for this review.

**Discussion:**

Since the WHO endorsed the use of PPPs as a means of achieving UHC, PPPs in the health sector have increasingly become popular for many healthcare projects and services especially in SSA. Yet, limited evidence around how PPP models have taken shape and their impact on achieving UHC creates a significant gap in understanding how PPPs affect progress toward UHC.

**Conclusion:**

To the best of our knowledge, this scoping review is the first to identify and examine healthcare PPPs in SSA for UHC. The approaches outlined in this protocol make several important advances in methods for examining PPPs, particularly for UHC in SSA, and can be applied elsewhere.

**Protocol Registration:**

This protocol has been registered on Open Science Framework and can be accessed at https://doi.org/10.17605/OSF.IO/V9874.

## 1 Introduction

Public–private partnerships (PPPs) in the health sector are contractual agreements between public and the private sector entities to pool resources and expertise with the aim of creating an effective, efficient, and responsive public health sector [1, 2, 3]. Governments worldwide have implemented PPPs, making significant contributions to the development and implementation of national health policies and strategies to improve health systems and health outcomes [4, 5].

Under PPPs, the public sector (also referred in this paper as governments) retains the responsibility as steward of the health sector and promulgator of policies and standards, as well as oversees compliance with the terms of PPPs to achieve public health goals [6, 7]. Further, funding of public health services continues to be provided by governments in the form of loan repayment of capital investment by the private sector, grants, and/or reimbursement for health services provided by the private sector under PPPs [8]. The private sectors, usually including religious organizations, corporations such as pharmaceutical companies and manufacturers, and philanthropists or private donor organizations, are engaged by the public sector with the aim to increase efficiency and quality of public health service delivery [7, 9, 10]. The private sectors play a significant role in health service delivery [11, 12]. They contribute to strengthening the supply chain for medicines, vaccines, medical supplies and consumables in both public and private health facilities [11, 12]. The private sectors also support health infrastructure development, encompassing hospitals, high‐end technology like magnetic resonance imaging (MRI), and cold chain management for life‐saving health commodities such as vaccines [13, 14]. Other key contributions include but are not limited to training, research, and capacity building, innovative health insurance models, and financial investment in the health sector [15].

The use of PPPs aligns with the World Health Organization's resolution [16] on universal health coverage (UHC), which seeks to ensure access to high‐quality essential healthcare services for all people, where and when needed. A key pillar of the UHC resolution is that all WHO member states reform their health‐financing systems by reducing out‐of‐pocket (OOP) health expenses via prepayment systems and ensuring equitable distribution of good‐quality healthcare infrastructure and human resources for health [16]. To achieve this, the WHO recommends governments (where appropriate) to collaborate with private providers and health‐financing organizations [16].

Despite commitments made by governments to attain UHC, most countries fall significantly short of UHC goals, particularly in sub‐Saharan Africa (SSA) [4]. For instance, in terms of health financing, there is a 30% average OOP health expenditure in SSA compared with 14% in Europe, 25% in East Asia and Pacific, and 10% in North America [17]. Further, UHC service coverage index in SSA shows a low rate at 43% [18]. Thus, more than half (57%) of people in SSA do not have access to essential healthcare services predominantly due to high OOP health expenditure [19, 20, 21]. In response, healthcare PPPs have emerged as an innovative policy option for SSA countries to achieve UHC [4, 22, 23, 24]. Several governments in SSA are adopting PPPs to build and/or manage public hospitals, install and/or operate high‐cost innovative technologies such as advanced diagnostic imaging, radiotherapy, and hemodialysis machines, or deliver specialized services to improve public healthcare delivery [23, 25].

Although the use of PPPs in the health sector is endorsed by governments in SSA [23], the evidence about their outcomes is mixed [26, 27]. For instance, several studies point to a range of positive outcomes associated with healthcare PPPs in improving both financial and physical access to healthcare services in deprived areas [28]. Hellowell [25] and Pinz et al. [29] argue that healthcare PPPs help to achieve optimum efficiency and cost‐effectiveness of health resources [25, 29]. Other evidence reveals that healthcare PPPs facilitate equitable access to health services, especially those not available in public health facilities, without financial impoverishment, particularly for the vulnerable populations who seek these services [1, 27, 30, 31]. However, there is also evidence of poor outcomes in terms of the quality and affordability of health services provided, the cost‐effectiveness of PPP projects, geographical access, and institutional challenges associated with healthcare PPPs [23, 25, 29, 32, 33, 34].

Despite the large body of literature available on the impact of healthcare PPPs, it appears no review has been conducted to synthesize evidence on how the use of PPPs in the health sector affects progress towards attaining UHC in SSA. For instance, a scoping review conducted by Joudyian et al. [21] examined evidence on the use of PPPs in providing primary healthcare (PHC) services but not specifically in the context of SSA. Other previous reviews on healthcare PPPs [35, 36, 37, 38, 39], including those conducted in SSA [2, 3, 40], have not synthesized literature on healthcare PPPs and the evidence available does not focus on how they aid in achieving UHC in SSA. This review seeks to fill this research gap by systematically synthesizing the literature on healthcare PPPs used in SSA and examining how various forms of healthcare PPPs affect efforts to achieve UHC in SSA.

Undertaking a scoping review will help to clarify broad areas of the concept [41], healthcare PPPs. Moreover, it will help to explore the extent of literature on healthcare PPPs and map evidence on the various forms of healthcare PPPs [42] and how they aid the attainment of UHC in SSA.

### 1.1 Theoretical Approach: Healthcare PPPs

#### 1.1.1 Classification of PPPs in the Health Sector

The implementation of PPPs in the health sector is highly contextual and often country‐specific [43, 44]. Depending on their use within the health sector, various classifications have been made [5]. Some scholars, such as Reich [45], classify PPPs in the health sector based on the kind of partners involved, partners' shared goals and objectives to create social value, and partners' mutual agreement to harness resources and expertise. Similarly, Mitchell [46] also proposes four underlying factors including the scope of the PPP (local, national, or global level), the nature of the partners, the objective for PPP, and partners' level of commitment to the partnership. Based on the two aforementioned studies as well as classification by other scholars [1, 28, 47, 48, 49], PPP models in the health sector exist along a continuum that ranges from health infrastructure development to health service delivery within the scope and objectives of the PPP.

#### 1.1.2 Healthcare PPP Models

Several models for categorizing healthcare PPPs have been developed [43]. The majority of models [5, 43, 44, 49, 50] categorize healthcare PPPs into three broad dimensions: health services PPPs, health infrastructure PPPs, and integrated PPPs. Briefly, infrastructure‐based PPPs relate to the design, build, and/or operate health infrastructure such as public health facilities. Service delivery PPPs focus on contracting, franchising, and/or management of public health services including, high‐end radio‐diagnostics and specialty clinical services, whereas integrated PPPs combine the functions of health services and infrastructure‐based PPPs (Table 1). Integrated PPPs include but are not limited to building hospitals, installing and/or managing medical equipment such as MRI, radiotherapy and hemodialysis machines, and medicines by the private sector [49].

**Table 1:** Typologies of healthcare PPP models used worldwide.

Authors/PPP model	Dimensions	Variations
Stucke and Humphreys [44] Abuzaineh et al. [49] World Bank Group [5] World Health Organization [43] Rita [51] Barlow et al. [50]	Health services/clinical services model	Contracting in Outsourcing Co‐location Contracting out Management contract Franchising
	Health infrastructure/infrastructure‐based model	Design build finance maintain (DBFM), Design build finance maintain operate (DBFMO) Design build operate transfer (DBOT) Private finance initiative (PFI) concession
	Integrated model (services plus infrastructure)	Variants of the infrastructure‐based PPPs including hospitals, high‐cost innovative technologies like MRI, hemodialysis and radiotherapy machines plus clinical and/or nonclinical services.
Raman and Björkman [1]	Financial protection PPPs	Health insurance Vouchers Service coupons Health cards Conditional cash transfers Cash incentive
	“Other models”	PPM Social marketing Telemedicine Training, research, and capacity building Regulation and governance Networks/alliances

*Note:* Raman and Björkman's [1] model includes the health service PPPs, infrastructure‐based PPPs/integrated PPPs, financial protection PPPs, and “other models”.

The model put forward by Raman and Björkman [1] adds two additional and important dimensions to this list. Firstly, Raman and Björkman's [1] model highlights an additional dimension known as financial protection PPPs, a critical element for UHC not incorporated in other models [5, 43, 44, 49, 50]. Financial protection PPPs are purchasing arrangements where health services are provided by accredited private health providers for free, or at a reduced cost, using purchase instruments, such as insurance or vouchers (Table 1). These forms of partnerships aim to protect the poor and vulnerable populations from financial risk when accessing quality health services [28]. Secondly, their model [1] includes another dimension known as “other models” which integrates all forms of partnerships not classified under any of the dimensions mentioned above. This includes the public–private mix (PPM) for surveillance, diagnosis, testing, and/or treatment of diseases, telemedicine, training, research, and capacity building, regulation and governance, networks and alliances, and the social marketing PPPs for health promotion. Thus, Raman and Björkman's [1] model allows for a holistic examination of PPPs in the health sector, particularly for UHC.

**Figure 1:**
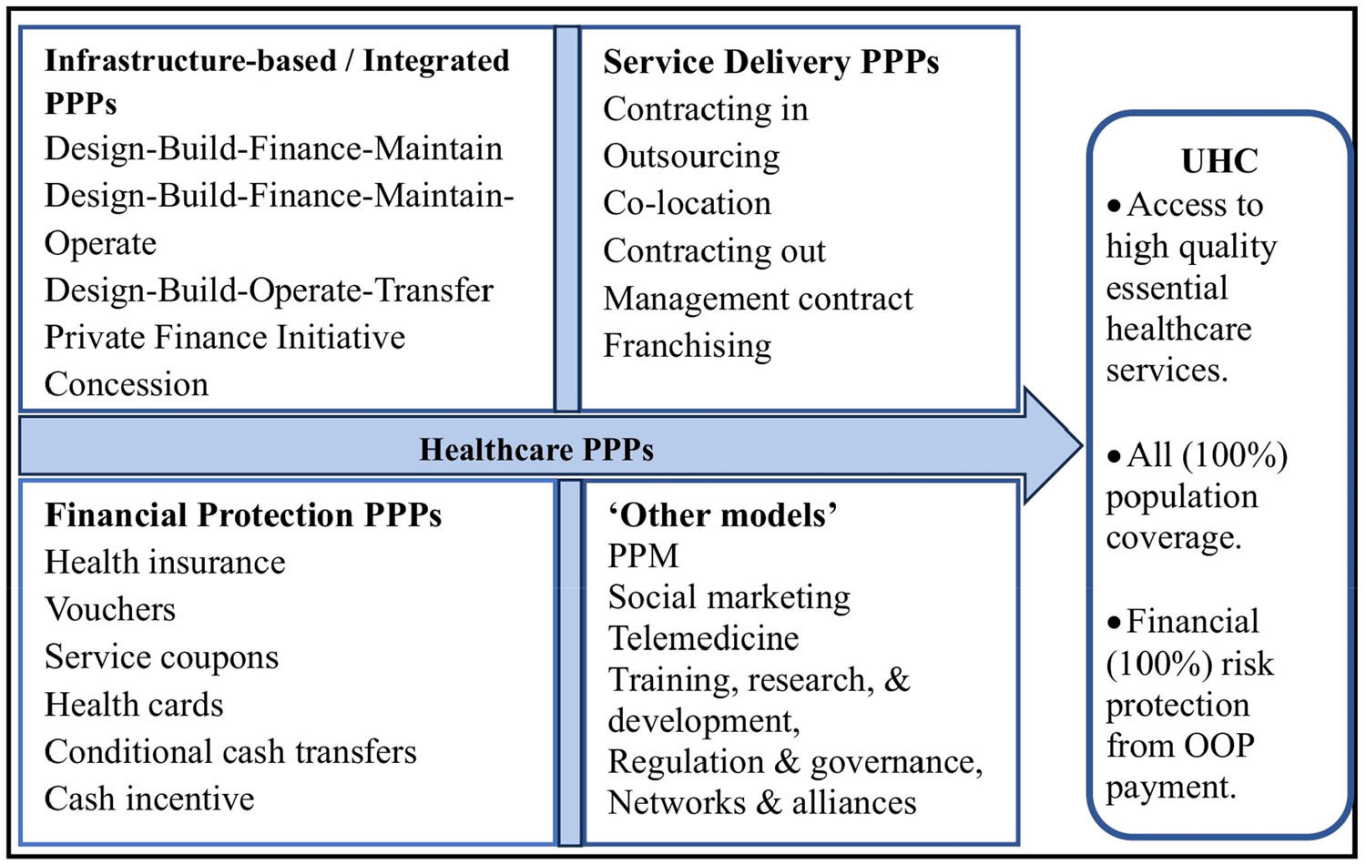
Healthcare PPPs for UHC. *Source:* Authors' design from PPP models in the healthcare sector by Raman and Björkman's [1].

To facilitate a comprehensive exploration of forms of PPPs in the health sector, this scoping review will adopt the healthcare PPP model espoused by Raman and Björkman [1]. In doing so, this review will map existing literature on healthcare PPPs in SSA onto the four‐stream PPP model (Figure 1), examine the level of access, population coverage and affordability of health services, and identify key facilitators and barriers to implementing healthcare PPPs in SSA.

## 2 Methods

### 2.1 Study Design

This review will include qualitative, quantitative, and/or mixed methods studies on healthcare PPPs in SSA. Arksey and O'Malley's [52] guidelines for conducting scoping reviews will guide the review process. The guidelines involve five stages: (1) identifying research questions, (2) identifying relevant papers, (3) selecting studies, (4) extracting the data, and (5) collating, summarizing, and reporting findings. The Preferred Reporting Items for Systematic Reviews and Meta‐Analysis extension for Scoping Reviews (PRISMA‐ScR) will be employed to present the review findings [53].

#### 2.1.1 Stage 1. Identifying Research Questions

**Figure 2:**
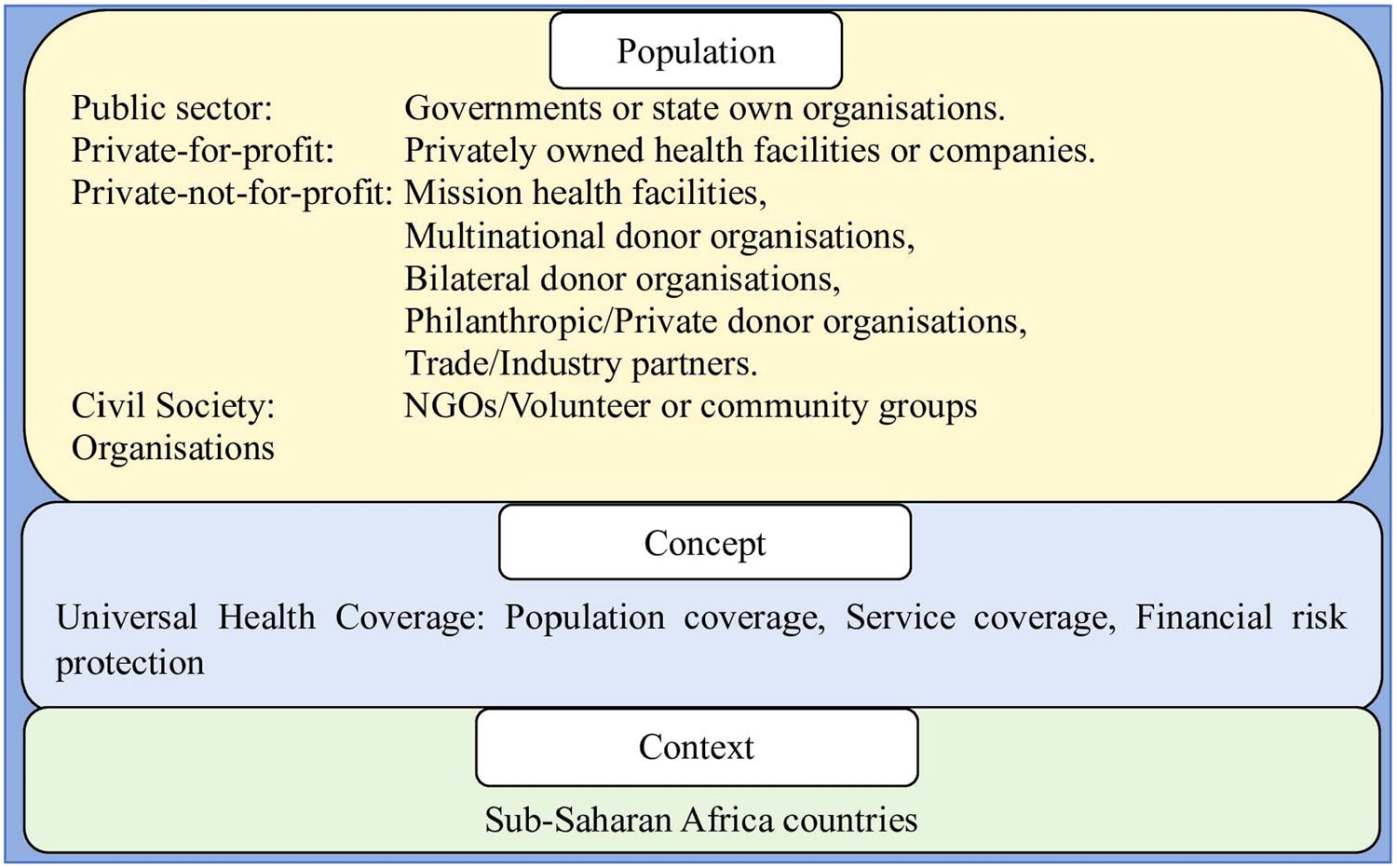
Population concept context framework.

The population, concept and context (PCC) framework [54] informed the research questions (Figure 2). The population refers to public partners, private partners, and Civil Society Organisations (CSOs) at all levels including local, national, and global. The public partners include governments or state‐owned organizations. Private partners, on the other hand, entail private not‐for‐profit (PNFP) and private for‐profit (PFP) organizations. PNFP organizations include mission health facilities, and multinational, bilateral, and philanthropists/private donor organizations, and trade/industry partners (such as manufacturers, telecommunication, mining companies, garment industries, research/consultancy companies) participating in health service activities. PFP organizations are mainly privately owned health facilities or companies. CSOs include nongovernmental organizations (NGOs) and volunteer or community groups. The concept is UHC within the SSA context. The scoping review seeks to address the following research questions:

i. What are the models or forms of healthcare PPPs for UHC in SSA?
ii. How do these models or forms of healthcare PPPs affect access, population coverage, and affordability of health services in SSA?
iii. What are the facilitators and key barriers to implementing these models or forms of healthcare PPPs, and how do they affect progress toward achieving UHC?

#### 2.1.2 Stage 2. Identifying Relevant Papers

This review will adopt a three‐step search strategy for scoping reviews as recommended by the Joanna Briggs Institute [55] to ensure a comprehensive search. The first step entails conducting an initial search, which has been done on PubMed with the help of an experienced subject librarian at the University of Otago. This was done by assessing the titles, abstracts, and indexed terms and modifying the search terms to cover a broader area of healthcare PPPs in SSA. The final search strategy has been developed for the PubMed database (Table 2). Secondly, the search strategy developed for PubMed will be applied to five other electronic databases: Medline (Ovid), Global Health (Ovid), Web of Science, Scopus, and EconLit. Lastly, a manual search through the reference lists of all eligible articles will be conducted for additional relevant studies. The search will be limited to peer‐reviewed articles (excluding grey literature) on healthcare PPPs from January 2013 to December 2023. The search restriction, as suggested by Arksey and O'Malley [52], is due to practical issues, such as time and resources, which point to the need to limit the scope of papers to be included.

**Table 2:** Final PubMed search result.

Search	Query	Outcome
#1	Public‐private partnerships OR public‐private participation OR public‐private collaboration OR public‐private engagement OR public‐private collaboration OR public‐private engagement OR public‐private partnerships OR public‐private participation OR public‐private collaboration OR public‐private engagement OR public‐private mix	23,302
#2	universal health coverage OR national health insurance OR social health Insurance OR community‐based insurance OR health services accessibility OR health care access OR health care cost OR health care finance OR health finance	988,695
#3	sub‐Saharan Africa OR Eastern Africa OR Western Africa OR Central Africa OR Southern Africa	311,233
#4	#1 AND #2 AND #3	665

#### 2.1.3 Stage 3. Selecting Studies

All article citations retrieved from the six electronic databases and from the manual search will be exported to EndNote library. After, duplicate citations will be removed electronically using the Endnote “find duplicate” function. This will be followed by a title and abstract screening by two reviewers. Afterward, a full‐text screening by the two reviewers will be done to select eligible relevant papers by applying the inclusion and exclusion criteria (Table 3). In case of disagreement, three reviewers will be consulted.

**Table 3:** Inclusion and exclusion criteria.

Inclusion	Exclusion
Published peer‐reviewed English language articles from January 2013 to December 2023. Publications relating to healthcare PPPs for achieving UHC in SSA. Publications that focus either on the models/forms of healthcare PPPs, and/or facilitators and/or barriers to achieving UHC in SSA. All forms of healthcare PPPs at either the local, national, and/or global level for UHC in SSA. PPPs in the health sector under formal and/or informal (mutual) agreements toward the achievement of UHC.	Publications in any language other than English and not published between January 2013 and December 2023. Publications relating to PPPs in the health sector but not in the SSA context. Grey literature on healthcare PPPs (such as non‐peer reviewed articles, theses, reports, unpublished documents, and unpublished manuscripts). PPPs not within the health sector for the purpose of ensuring UHC.

#### 2.1.4 Stage 4. Extracting the Data

A data extraction form will be designed on Microsoft Excel spreadsheet with the following column headings: author/publication year, country, objectives, methods, name of PPP, partners/PPP arrangements, services/intervention for UHC, and key findings. The form will be used to collect and sort relevant information from selected articles.

#### 2.1.5 Step 5. Collating, Summarizing, and Reporting Findings

Firstly, various forms of healthcare PPP identified from the selected studies will be mapped onto the healthcare PPP model by Raman and Björkman [1] along the three core dimensions of UHC [56] (Table 4). After, thematic analysis of data will be conducted by identifying recurring themes in the selected articles and categorizing them under a thematic heading [57]. A coding framework will be developed to describe the facilitators and barriers reported from the use of healthcare PPPs in SSA to describe how they affect progress towards achieving UHC.

**Table 4:** Mapping healthcare PPPs in SSA towards UHC onto the healthcare PPP model.

Dimensions of PPPs	Variations under each dimension	Country	UHC elements		
			SC	PC	FRP
Infrastructure‐based PPPs	—	—	—	—	—
Integrated PPPs	—	—	—	—	—
Service delivery PPPs	—	—	—	—	—
Financial protection PPPs	—	—	—	—	—
“Other models”	—	—	—	—	—
Total number of studies			—	—	—

Abbreviations: FRP, financial risk protection; PC, population coverage; SC, service coverage.

### 2.2 Potential Limitations and Strengths

The search restrictions regarding language, publication date and paper type, may exclude other high‐quality literature on healthcare PPPs in SSA which may increase the potential for selection bias in identifying literature for the review [58]. Further, authors recognize the fact that scoping review methods are less rigorous than systematic reviews as no quality assessment of included studies is conducted [41]. However, employing the methodological guidance [52] and the reporting criteria [53] as well as applying the eligibility criteria will minimize the risk of error, and thus enhance the quality and credibility of the review findings [59]. Moreover, using findings from different data sources and methods will improve the validity of the review findings [60].

## 3 Discussion

Since the WHO endorsed the use of PPPs as a means of achieving UHC [16], PPPs in the health sector have increasingly become popular for many healthcare projects and services, especially in SSA [23, 28]. Yet, limited evidence around how PPP models have taken shape and their impact on achieving UHC creates a significant gap in understanding how PPPs affect progress toward UHC. By synthesizing evidence on existing healthcare PPPs in SSA and how they aid the achievement of UHC, this review will help to identify key knowledge gaps and provide valuable insights and direction for further research and policymaking in SSA and elsewhere.

## 4 Conclusion

To the best of our knowledge, this scoping review is the first to identify and examine healthcare PPPs in SSA, highlighting all forms of partnerships in the health sector towards the attainment of UHC. This review will facilitate a more comprehensive understanding of healthcare PPPs, incorporating insights from community to global‐level PPPs than has previously been achieved in other literature [2, 3, 35, 36, 37, 38, 39, 40]. Further, it presents a novel framework for mapping all forms of partnerships in the health sector along the three core elements of UHC to assess how the various forms of PPPs impact UHC. The approaches outlined in this protocol make several important advances in methods for examining PPPs, particularly for UHC in SSA, and can be applied elsewhere.

## Author Contributions

**Godfred Otchere:** conceptualization, methodology, investigation, writing – original draft, writing – review and editing, formal analysis. **Adam Fusheini:** investigation, writing – original draft, methodology, writing – review and editing, formal analysis. **Trudy Sullivan:** writing – original draft, writing – review and editing, formal analysis. **Robin Gauld:** writing – original draft, writing – review and editing, formal analysis. **Erin Penno:** writing – original draft, methodology, writing – review and editing, formal analysis, investigation.

## Ethics Statement

The authors have nothing to report.

## Conflicts of Interest

The authors declare no conflicts of interest.

## Transparency Statement

The lead author Godfred Otchere affirms that this manuscript is an honest, accurate, and transparent account of the study being reported; that no important aspects of the study have been omitted; and that any discrepancies from the study as planned (and, if relevant, registered) have been explained.

## Data Availability

The authors have nothing to report.
